# Genome Wide Expression Profiling Reveals Suppression of Host Defence Responses during Colonisation by *Neisseria meningitides* but not *N. lactamica*

**DOI:** 10.1371/journal.pone.0026130

**Published:** 2011-10-20

**Authors:** Hazel En En Wong, Ming-Shi Li, J. Simon Kroll, Martin L. Hibberd, Paul R. Langford

**Affiliations:** 1 Infectious Diseases, Genome Institute of Singapore, Singapore, Singapore; 2 Section of Paediatrics, Imperial College London, London, United Kingdom; Health Protection Agency, United Kingdom

## Abstract

*Both Neisseria meningitidis* and the closely related bacterium Neisseria lactamica colonise human nasopharyngeal mucosal surface, but only *N. meningitidis* invades the bloodstream to cause potentially life-threatening meningitis and septicaemia. We have hypothesised that the two neisserial species differentially modulate host respiratory epithelial cell gene expression reflecting their disease potential. Confluent monolayers of 16HBE14 human bronchial epithelial cells were exposed to live and/or dead *N. meningitidis* (including capsule and pili mutants) and *N. lactamica*, and their transcriptomes were compared using whole genome microarrays. Changes in expression of selected genes were subsequently validated using Q-RT-PCR and ELISAs. Live *N. meningitidis* and *N. lactamica* induced genes involved in host energy production processes suggesting that both bacterial species utilise host resources. *N. meningitidis* infection was associated with down-regulation of host defence genes. *N. lactamica*, relative to *N. meningitidis*, initiates up-regulation of proinflammatory genes. Bacterial secreted proteins alone induced some of the changes observed. The results suggest *N. meningitidis* and *N. lactamica* differentially regulate host respiratory epithelial cell gene expression through colonisation and/or protein secretion, and that this may contribute to subsequent clinical outcomes associated with these bacteria.

## Introduction

*Neisseria meningitidis* and *Neisseria lactamica* are commensal bacteria that colonise the mucosal surface of the human nasopharynx. On rare occasions, *N. meningitidis* can enter the bloodstream and cause invasive disease with a reported incidence from 1–3 per 100,000 cases in industrialised countries [1], whilst *N. lactamica* does not cause invasive disease. *N. lactamica* is associated with colonisation of the nasopharynx in the first few years of life which wanes with age, the converse being found with *N. meningitidis* [2], [3], [4]. Hence, it has been suggested that colonisation with *N. lactamica* can protect against meningococcal disease, supported by recent studies which show that carriers of *N. lactamica* develop cross-reacting opsonophagocytic antibodies to *N. meningitidis* [5]. Colonisation by *N. meningitidis* involves the adherence of host epithelial cells, which is mediated by components such as the pili [6].

Although *N. meningitidis* and *N. lactamica* are closely related bacteria, with 60% similarity in their genomes [7], there are important gene differences between the two neisserial species which affect their interactions with the host. For example, the genes required for capsule expression are present in *N. meningitidis* but not *N. lactamica* [7]. The capsule can protect *N. meningitidis* against phagocytosis [8], complement mediated lysis [9] and prevent desiccation during transmission [10], and is considered an important virulence factor. The presence of a capsule in meningococci reduces both adherence and invasion of nasopharyngeal epithelial cells by masking adhesins and invasins [11]. Mutation of the meningococcal *pilE* gene, encoding the major pilin subunit, results in greatly reduced adherence of bacteria to endothelial and epithelial cells [12]. It is not known whether an equivalent mutation in *N. lactamica* would have similar effects. In contrast to the amount of data available on the role of microbial factors in adherence and invasion of eukaryotic cells, there is a lack of information on the comparative host response, in particular with respiratory tract epithelial cells, to *N. meningitidis* and *N. lactamica*. A recent review by Schubert-Unkmeir et al. [13] outlines the various human gene expression studies that have been done so far using cell lines other than those representing the respiratory tract in response to *N. meningitidis*. Microarrays (comprising a limited number of inflammation, adhesion and iron-homeostasis genes) were used to investigate the host response of ME-180 epithelial-like human cervical carcinoma [14] and A431 human epidermoid carcinoma cells [15] to *N. meningitidis*. Bonnah et al. [14] showed that the mRNA expression of several host genes involved in iron homeostasis were altered upon infection with meningococci, while Plant et al. [15] showed that there was an induction of chemokine receptors and cytokines such as CXCR-4, CXCR-5, IL1A, IL1B, IL18 and IFN-γ , with most of the host genes induced early in infection.

Other microarray studies have investigated the responses of human endothelial cells to *N. meningitidis*. Using primary human umbilical vein endothelial cells, Linhartova et al. [16] have shown that pilus-mediated adhesion and growth of meningococci in microcolonies on the host cell surface results in alteration of expression levels of human genes known to regulate apoptosis, cell proliferation, inflammatory response and adhesion, and of genes for signalling pathway proteins such as TGF-β/Smad, Wnt/β-catenin and Notch/Jagged. It was suggested that the response found increased the ability of host cells to withstand apoptotic signals induced by infection, thus allowing the maintenance of normal cell function, and subsequently bacterial colonisation. A human cDNA microarray of 11,835 genes was used to study the response of human brain microvascular endothelial cells to *N. meningitidis* [17]. Host genes involved in apoptosis, cell adhesion, downstream signalling of integrins (and their negative regulators) and cytoskeleton reorganisation were significantly differentially regulated at 4 and 8 hours post infection. The influence of capsule on host gene expression was investigated by comparing the host response to wild type (WT) MC58 strain with that in response to an isogenic *siaD* knockout mutant (also known as a *cap*- mutant), which does not possess a capsule. At 4 and 8 hours post infection, the expression of 49.4% and 45% of host genes, respectively, were considered to be capsule-dependent. Response to *N. meningitidis* in whole blood has also been investigated using custom-printed cDNA microarrays consisting of about 18,000 genes, with the aim of identifying a serum factor causing cardiac dysfunction in meningococcal septic shock [18]. Two studies have described the response of human meningothelial cells to *N. meningitidis* and its secreted proteins. Using human broad range cDNA expression arrays for 3528 genes, Wells et al. [19] observed an up-regulation of proinflammatory cytokines such as IL-6 and IL-8, as well as of anti-apoptosis genes in meningothelial cells, suggesting that genes involved in immunity and defence are activated in response to *N. meningitidis*, but at the same time the host cells are able to resist the damaging effects of the bacteria. A follow up study by Robinsonet al. [20] using a microarray comprising of cytokine and apoptosis genes (573 in total) showed that secreted protein preparations from *N. meningitidis* induced host pro-inflammatory responses and resistance to apoptosis, suggesting that secreted proteins are important in meningococcal-host interactive biology.

More recently, there have been studies reported that have investigated the host response of respiratory tract epithelial cells to *N. meningitidis*, *N. lactamica* or components derived from them. Liu et al. [21] investigated the response of BEAS-2B human bronchial cells to purified PorB, a major outer membrane protein present in both *N. lactamica* and *N. meningitidis*. The *N. lactamica* PorB had a different Toll like receptor 2 (TLR2) binding specificity to that from the meningococcus. Compared to the PorB of *N. meningitidis*, the one from *N. lactamica* was a poorer inducer of proinflammatory mediators and of TLR2 expression in human airway epithelial cells, an effect also seen with live *N. lactamica*. With the nasopharyngeal cell line Detroit 562, Tezera et al. [22] found that *N. lactamica* induced a weak inflammatory response via attenuation of secretory cytokines such as TNF-α and IL-6, and to a lesser extent chemokines such as IL-8 and RANTES, compared to *N. meningitidis*. These authors have concluded that through TLR1/2 stimulation, by activating PPARγ and inhibiting NFκβ activity, *N. lactamica* plays an important role in suppressing pathogen-induced inflammation in the nasopharyngeal mucosa. Both of these studies were confined to a limited set of genes and focused on late time points, typically at 24 hours post infection.

In this study we have compared the transcriptomes of 16HBE14 human bronchial epithelial cells at time points 0 to 7 hours in response to *N. meningitidis* and *N. lactamica* as a surrogate model for the initial stages of respiratory tract colonisation. Both bacteria have been shown to associate with 16HBE14 cells [23]. Unlike in the previous microarray studies which have analysed a comparatively limited set of human genes, the Illumina HumanRef-8 BeadChip covering most of the whole human genome, representing 20,589 genes, was used. We hypothesise that early interactions of *N. meningitidis* and *N. lactamica* with respiratory epithelial cells are characterised by differential gene expression and this affects subsequent outcomes. Host gene expression profiles in response to both live and dead WT *N. lactamica* and *N. meningitidis*, *N. meningitidis* capsule (*cap*-) and pili (*pilE*-) mutants and secreted protein preparations from both bacteria were compared. In particular, using dead bacteria as a comparator, we have focused on host responses resulting from active bacterial processes since meningococcal gene expression, including those involved in processes such as host cell adhesion, amino acid and DNA metabolism is known to change after interaction with epithelial cells [24]. It has also been shown that when in contact with epithelial cells, *N. meningitidis* adds phosphoglycerol to its type IV pili and this posttranslational modification mediates a regulated detachment of the bacterium from the host, which is thought to facilitate its dissemination [25].

Our results suggest that both *N. meningitidis* and *N. lactamica* actively interact with respiratory tract epithelial cells and utilise host resources for energy, perhaps as a means of adaptation and colonisation. In addition, the data indicate that *N. meningitidis* down-regulates host defence genes whilst *N. lactamica* induces a proinflammatory response, suggesting specific colonisation processes that may lead to different clinical outcomes. Neisserial secreted proteins appear to be mediators of some of these differential host gene expression changes, suggesting novel mechanisms for modulation of the host response.

## Results

### Neisserial association and invasion of 16HBE14 bronchial epithelial cells

**Figure 1:**
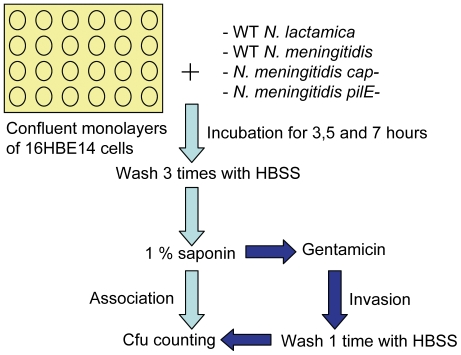
Experimental design to determine the association and invasion of *Neisseria* with 16HBE14 cells. 16HBE14 cells were seeded into 24-well plates and incubated in media for at least 48 h at 37°C in an atmosphere containing 5% CO_2_. Suspensions of wild type (WT) *N. lactamica* and *N. meningitidis*, *N. meningitidis cap*- and *pilE*- mutants in fresh media were each added separately to confluent epithelial cells with a multiplicity of infection of 30 and incubated for 3, 5 and 7 hours. At each time point, cells were either washed with Hanks' Balanced Salt Solution (HBSS) and lysed with saponin to count the number of bacteria associated with the cells, or incubated with gentamicin for 1 hour before washing and lysing with saponin to count the number of bacteria within the cells. Cfu: colony forming units.

**Figure 2:**
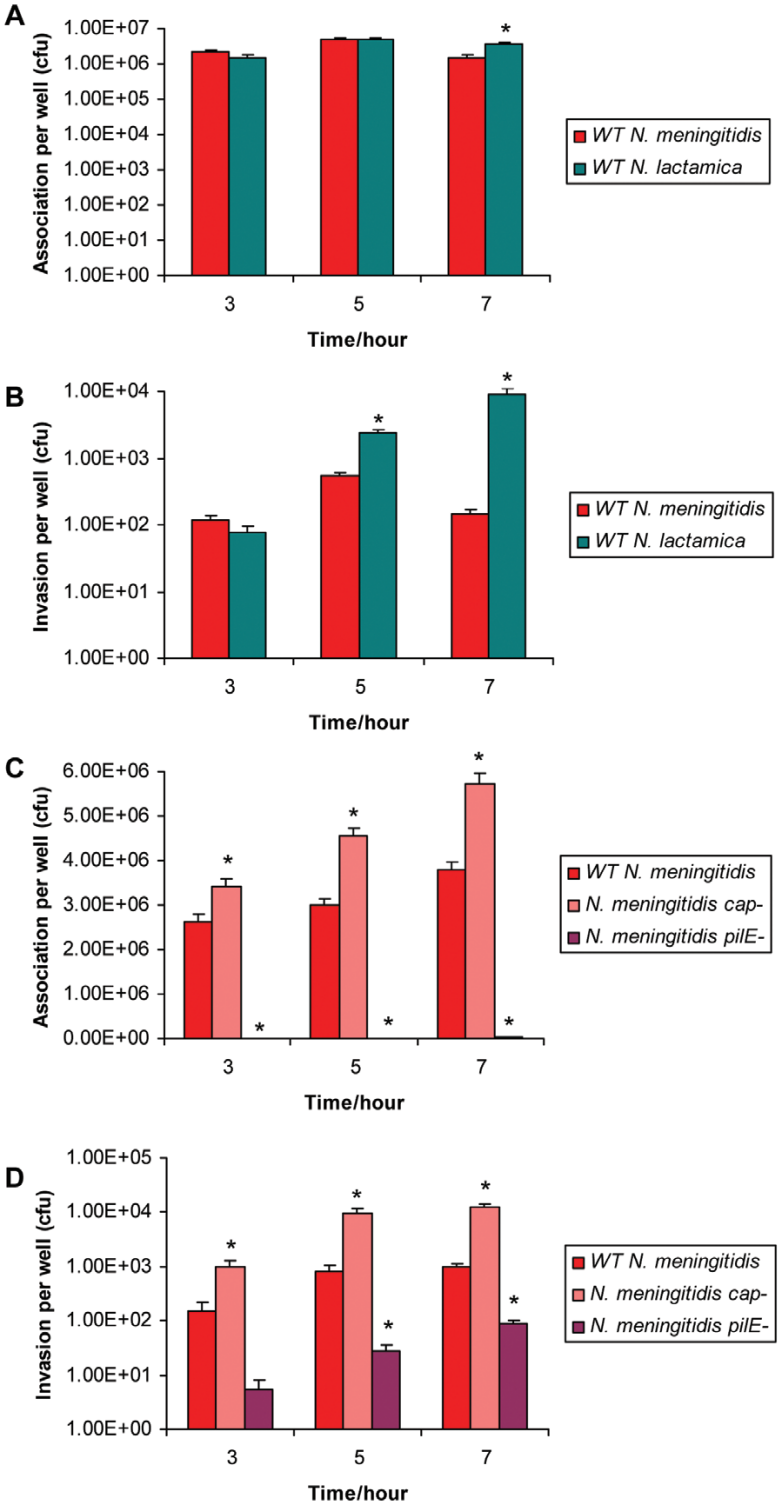
Comparing the association and invasion of *N. lactamica* and *N. meningitidis* with 16HBE14 cells. 16HBE14 cells were incubated with *N. meningitidis* and *N. lactamica* and association and invasion assays were carried out at various time points at 3, 5 and 7 hours. (A) and (B) compare the association and invasion of wild type (WT) *N. meningitidis* and *N. lactamica* respectively while (C) and (D) compares the association and invasion of WT *N. meningitidis* with the *cap*- and *pilE*- mutants, respectively. Values of colony forming units (cfu) per well represent means from at least 3 biological replicates with error bars indicating standard error of the mean. Asterisks (*) indicate statistical significance with a p-value of less than 0.05 compared to WT *N. meningitidis*.

To assist interpretation of transcriptome data, association and invasion assays were carried out as shown in Figure 1. These allowed the extent of interaction of WT *N. lactamica* and *N. meningitidis*, as well as *N. meningitidis cap*- and *pilE-* mutants with confluent monolayers of 16HBE14 human respiratory bronchial epithelial cells to be determined. The *N. meningitidis* serogroup B strain MC58, the genome sequence of which is known [26] and has been widely utilised in pathogenicity research [16], [17], [19], [20], was used. *N. lactamica* strain Y92-1009 was chosen as it has been evaluated as a vaccine strain for meningococcal disease [27] and its genome sequence has been determined [28]. There were no significant differences in association with epithelial cells between WT *N. lactamica* and *N. meningitidis* at 3 and 5 hours. However, significantly more *N. lactamica* associated at 7 hours (Figure 2A). While there was no significant difference in the numbers that invaded at 3 hours, significantly more *N. lactamica*, compared to *N. meningitidis*, invaded at 5 and 7 hours (Figure 2B). Association and invasion by the MC58 *cap*- mutant was significantly greater compared to the WT from 3 to 7 hours post infection (Figure 2 C and D). In addition, the association of the MC58 *pilE*- with epithelial cells was significantly less compared to the WT from 3 to 7 hours (Figure 2C), while the invasion by the MC58 *pilE*- was significantly less compared to the WT from 5 to 7 hours (Figure 2D).

### Host responses specific to live *N. meningitidis* occur at an earlier time point compared to those specific to live *N. lactamica*

**Figure 3:**
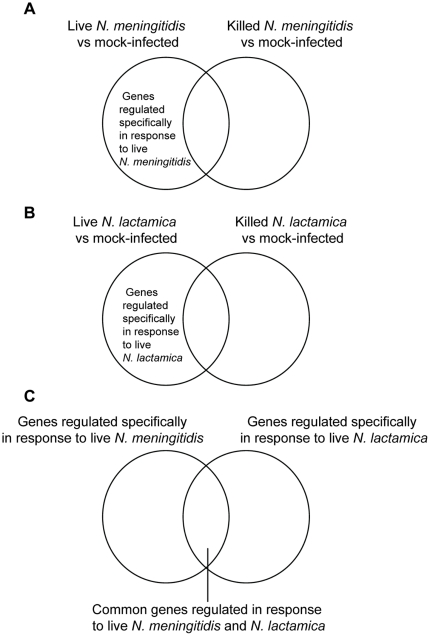
Identification of genes regulated specifically in response to live *Neisseria* determined by microarray analysis. Genes that were regulated specifically in response to live but not killed bacteria from 0 to 7 hours, for both *N. meningitidis* and *N. lactamica* were determined as follows: genes regulated in response to killed bacteria (compared to mock-infected controls) were subtracted from those regulated in response to live *N. meningitidis* (A) or *N. lactamica* (B) derived at each time point. These genes were then separated into three groups: those that were specific to *N. meningitidis*, those that were specific to *N. lactamica* and those that were common to both (C). These analyses were done for every time point up to 7 hours post infection.

Confluent monolayers of 16HBE14 bronchial epithelial cells were studied under the following conditions from 0 to 7 hours: mock-infected, infected with killed WT *N. lactamica*, with live WT *N. lactamica*, with killed WT *N. meningitidis* and with live WT *N. meningitidis*. Figure 3 shows how the lists of differentially expressed genes were compared to obtain genes regulated in response to live WT *N. meningitidis*, to live WT *N. lactamica* and common to both. Table 1 shows the number of genes in each group from 3 to 7 hours post infection. At an initial time point of 3 hours, there were 9 times more genes regulated specifically in response to live *N. meningitidis* (125 genes) compared to *N. lactamica* (14 genes). At 5 and 7 hours, however, the number of genes regulated specifically in response to live *N. lactamica* increased until it was almost comparable to those responding to *N. meningitidis*. At this same time point of 5 and 7 hours, there were also increased numbers of *N. lactamica* associated with and invading 16HBE14 cells (as shown in Figure 2 A and B). We next determined if there were any biological processes significantly over-represented by the genes regulated specifically in response to live WT *N. meningitidis*, to live WT *N. lactamica* and common to both, at each time point from 3 to 7 hours. The following sections describe these host cell responses determined by microarray, the expression of selected genes validated by quantitative real-time polymerase chain reaction (Q-RT-PCR), and in specific instances, by protein expression.

**Table 1:** Number of host genes regulated in response to live WT *Neisseria*.

Time/hour	Specific to *N. meningitidis*	Specific to *N. lactamica*	Common to both
3	125	14	8
4	204	425	66
5	186	198	106
6	247	103	90
7	220	185	132

### Host genes involved in metabolic and energy production processes were up-regulated in response to live N. meningitidis and N. lactamica at late time points

**Figure 4:**
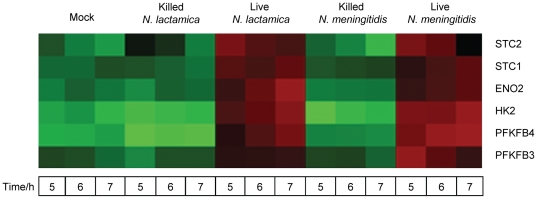
Microarray heat map of validated genes up-regulated in response to *N. lactamica* and *N. meningitidis*. The expression of the 6 validated genes from 5 to 7 hours is shown. Each column is the mean signal of 4 (*Neisseria* infected) to 8 replicates (mock-infected). Red indicates that the signal is higher relative to the rest of the samples while green indicates that the signal is lower.

**Figure 5:**
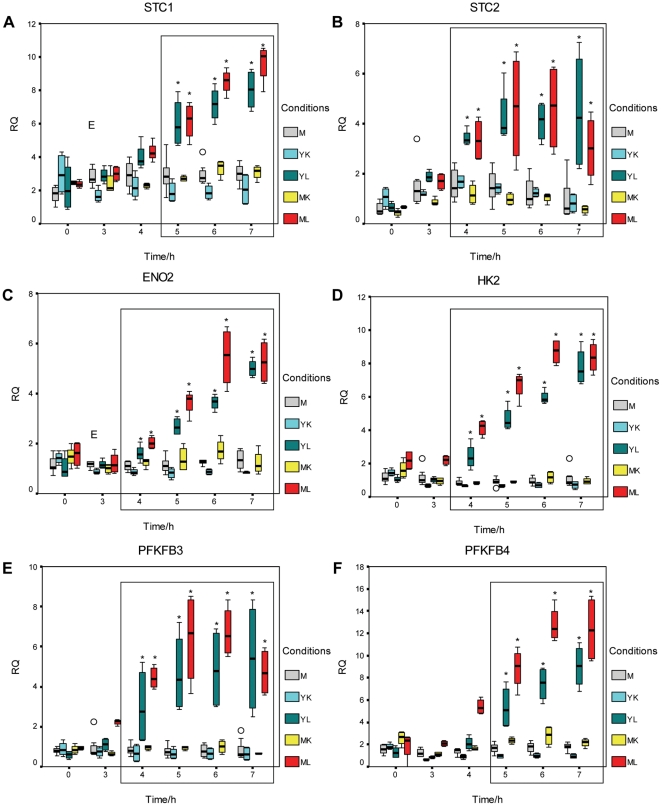
Q-RT-PCR of host genes up-regulated in response to both live *N. meningitidis* and *N. lactamica*. Expression at the transcript level is shown for STC1 (A) and STC2 (B), which are involved in the regulation of phosphate metabolism as well as ENO2 (C), HK2 (D), PFKFB3 (E) and PFKFB4 (F), which encode enzymes involved in glycolysis. There was significant up-regulation (with a p-value of less than 0.05) with respect to the mock-infected controls in response to both live but not killed *N. meningitidis* and *N. lactamica*. This occurred from 5 to 7 hours for STC1 (A) and PFKFB4 (F) and from 4 to 7 hours for STC2 (B), ENO2 (C), HK2 (D) and PFKFB3 (E) as indicated by the asterisks (*). M: mock-infected, YK: killed WT *N. lactamica*, YL: live WT *N. lactamica*, MK: killed WT *N. meningitidis*, ML: live WT *N. meningitidis*, RQ: relative quantification. An outlier (0) is defined as between 1.5 to 3× the interquartile range from the 25th or 75th percentile while an extreme data point (E) is defined as more than 3× the interquartile range from the 25th or 75th percentile.

Microarray analysis identified host genes that were regulated in response to both live WT *N. meningitidis* and *N. lactamica* and which clustered (using Panther software analysis) [29] into the biological process categories of phosphate metabolism (5 to 7 hours) and glycolysis (6 to 7 hours) (Table 2). The expression of the six genes representative of these biological processes is shown in a heat map in Figure 4, with their fold changes listed in Table S1. The time course expression of these six genes from 0 to 7 hours (using Q-RT-PCR) is shown in Figure 5. The genes are STC1 and STC2 (stanniocalcin 1 and 2), which are involved in the regulation of phosphate metabolism, ENO2 (enolase 2), HK2 (hexokinase 2), PFKFB3 and PFKFB4 (phosphofructokinases 3 and 4), which encode enzymes involved in glycolysis. Significant up-regulation of these genes compared to the mock-infected controls in response to both live WT *N. meningitidis* and *N. lactamica* occurred from 5 to 7 hours for STC1 and PFKFB4 and from 4 to 7 hours for STC2, ENO2, HK2 and PFKFB3, with expression increasing with time. None of these genes was activated upon addition of killed *N. meningitidis* or *N. lactamica*, indicating that the up-regulation was specific to live bacteria.

**Table 2:** Host biological processes associated with both live WT *N. meningitidis* and *N. lactamica*.

	Time points (hour)				
Biological Process	3	4	5	6	7
Regulation of phosphate metabolism	-	-	1.7E-2	1.3E-2	2.7E-2
Glycolysis	-	-	-	1.1E-2	4.8E-2

Significance of biological processes is expressed as p-values and dashes indicate no significance was identified at the time point.

### Immunity and defence genes were down-regulated in response to live *N. meningitidis* but not *N. lactamica* at 3 hours

**Figure 6:**
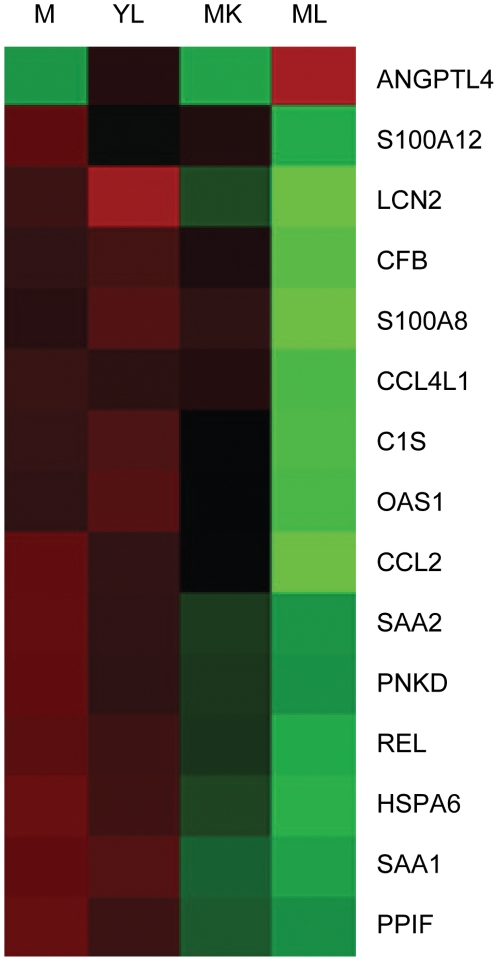
Microarray heat map of immunity/defence genes regulated specifically in response to live WT *N. meningitidis*. The expression of the 15 validated genes at 3 hours is shown. Each column is the mean signal of 4 (*Neisseria* infected) to 8 replicates (mock-infected). Red indicates that the signal is higher relative to the rest of the samples while green indicates that the signal is lower. M: mock, YL: Live WT *N. lactamica*, MK: Killed WT *N. meningitidis*, ML: Live WT *N. meningitidis*.

At 3 hours, there was a significant over-representation of genes in the immunity and defence category that were regulated specifically in response to live WT *N. meningitidis* (p = 3.8E-3). The expression of the genes validated by Q-RT-PCR at 3 hours is shown in a heat map in Figure 6, with their fold changes listed in Table 3. Fourteen out of 15 (93%) of genes in this category were down-regulated in response to live WT *N. meningitidis* (but not to *N. lactamica*).

**Table 3:** Validated immunity and defence genes regulated specifically in response to live WT *N. meningitidis* at 3 hours.

Human Genbank ID	Symbol	Description	*N. meningitidis* vs Mock	
			Array	Q-RT-PCR
NM_139314	ANGPTL4	angiopoietin-like 4	2.5	1.8
NM_001734	C1S	complement component 1, s subcomponent	−2.2	−2.5
NM_002982	CCL2	chemokine (C-C motif) ligand 2	−2.7	−3.2
NM_001001435	CCL4L1	chemokine (C-C motif) ligand 4-like 1	−2.2	−2.8
NM_001710	CFB	complement factor B	−2.2	−2.3
NM_002155	HSPA6	heat shock 70 kDa protein 6 (HSP70B')	−2.3	−1.7
NM_005564	LCN2	lipocalin 2	−3.0	−3.5
NM_016816	OAS1	2′,5′-oligoadenylate synthetase 1, 40/46 kDa	−2.1	−1.4
NM_022572	PNKD	paroxysmal nonkinesiogenic dyskinesia	−2.0	−1.7
NM_005729	PPIF	peptidylprolyl isomerase F (cyclophilin F)	−2.0	−1.9
NM_002908	REL	v-rel reticuloendotheliosis viral oncogene homolog (avian)	−2.1	−2.0
NM_005621	S100A12	S100 calcium binding protein A12	−2.2	−2.1
NM_002964	S100A8	S100 calcium binding protein A8	−2.7	−3.6
NM_199161	SAA1	serum amyloid A1	−2.1	−2.2
NM_030754	SAA2	serum amyloid A2	−2.1	−2.0

The microarray and Q-RT-PCR results show the fold changes of validated genes regulated in response to live WT *N. meningitidis* with respect to mock-infected controls.

**Figure 7:**
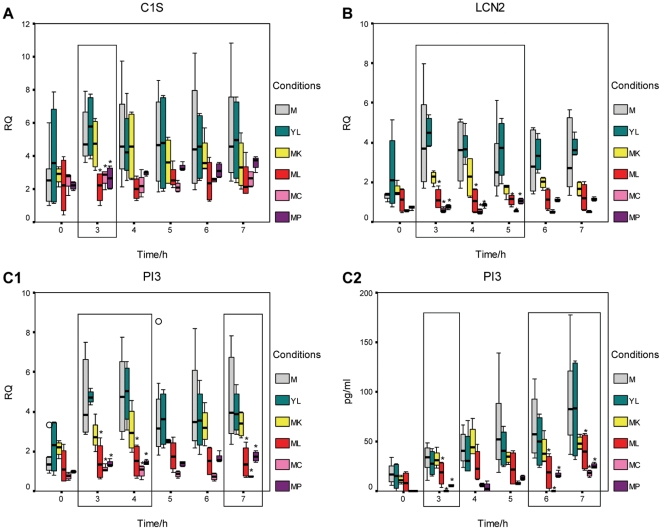
Expression of host genes down-regulated specifically in response to live WT and mutant *N. meningitidis*. The expression of C1S, LCN2 and PI3 was down-regulated specifically in response to live WT and mutant *N. meningitidis*. This occurred at a time point of 3 hours for C1S (A), from 3 to 5 hours for LCN2 (B) and at 3, 4 and 7 hours for PI3 (C1) at the transcript level using Q-RT-PCR. Measurement of PI3 at the protein level also indicated a down-regulation at 3, 6 and 7 hour time points (C2). Asterisks (*) indicate statistical significance with a p-value of less than 0.05 with respect to mock-infected samples. M: mock-infected, YL: live WT *N. lactamica*, MK: killed WT *N. meningitidis*, ML: live WT *N. meningitidis*, MC: *N. meningitidis cap*-, MP: *N. meningitidis pilE*-.RQ: relative quantification. An outlier (0) is defined as between 1.5 to 3× the interquartile range from the 25th or 75th percentile.

The 3 hour time point was the only one where genes that were regulated specifically in response to live WT *N. meningitidis* clustered into the immune and defence category. The loss of capsule and pili respectively enhances and reduces the association and invasion of *N. meningitidis* (Figure 2 C and D). Genes whose expression is altered in response to WT *N. meningitidis*, *N. meningitidis cap*- and *N. meningitidis pilE*- mutants (and so are independent of the capsule and pili) could be mediated by a live, contact-independent process such as secreted proteins. Therefore, we infected 16HBE14 cells with *N. meningitidis cap*- or *pilE*- mutants and identified host genes that were similarly differentially expressed in response to live WT and mutant *N. meningitidis*, compared to mock-infected controls, at 3 hours. Table 4 shows the fold changes of these genes found from the microarray and validated by Q-RT-PCR at 3 hours post infection. Five out of 6 (83%) of these genes were down-regulated with respect to mock-infected controls. The expression of 3 of these validated immune-related genes, C1S (complement component 1, s subcomponent), LCN2 (lipocalin 2) and PI3 (peptidase inhibitor 3) were followed up from 0 to 7 hours (Figure 7). C1S is a component of the complement pathway while LCN2 and PI3 are antimicrobial peptides. Down-regulation of C1S, LCN2 and PI3 (with respect to mock-infected controls) was specifically associated with live WT and mutant *N. meningitidis* (*cap*- and *pilE*-). This occurred at a time point of 3 hours for C1S (Figure 7A), from 3 to 5 hours for LCN2 (Figure 7B) and at 3, 4 and 7 hours for PI3 at the transcript level (Figure 7C1). Measurement of PI3 at the protein level also indicated a down-regulation at 3, 6 and 7 hour time points (Figure 7C2).

**Table 4:** Validated genes regulated similarly in response to WT and mutant *N. meningitidis* at 3 hours.

Human Genbank ID	Symbol	Description	WT *N. meningitidis*		*N. meningitidis cap*-		*N. meningitidis pilE*-	
			Array	Q-RT-PCR	Array	Q-RT-PCR	Array	Q-RT-PCR
NM_183376	ARRDC4	arrestin domain containing 4	−3.1	−2.8	−2.8	−2.4	−1.6	−1.9
NM_001734	C1S	complement component 1, s subcomponent	−2.2	−2.5	−1.8	−2.0	−1.4	−2.0
NM_005564	LCN2	lipocalin 2	−3.0	−3.5	−5.2	−6.9	−2.6	−5.4
NM_005764	PDZK1IP1	PDZK1 interacting protein 1	−4.4	−4.4	−76.9	−6.5	−14.3	−4.7
NM_002638	PI3	peptidase inhibitor 3	−3.4	−3.1	−4.6	−4.2	−2.4	−3.4
NM_002928	RGS16	regulator of G-protein signalling 16	2.5	1.8	4.3	4.2	3.2	2.1

The microarray and Q-RT-PCR results show the fold changes of the validated genes with respect to mock-infected controls.

### *N. lactamica* activates proinflammatory cytokines and genes encoding transcription factors and a cytoplasmic protein at higher levels compared to *N. meningitidis*

**Figure 8:**
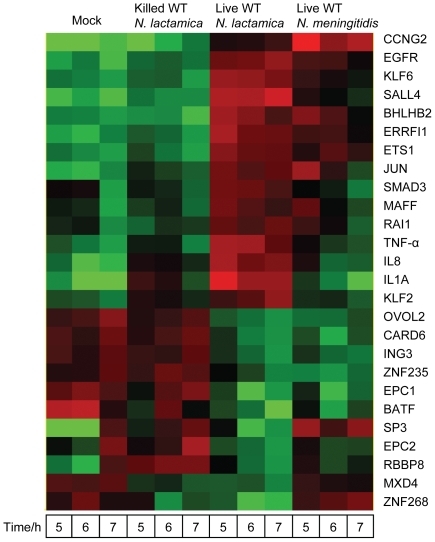
Microarray heat map of genes regulated specifically in response to live WT *N. lactamica*. The expression of the 26 validated genes clustering into significant biological processes is shown, with some genes activated more and for a longer period of time from 5 to 7 hours in response to live WT *N. lactamica* compared to live WT *N. meningitidis*. Each column is the mean signal of 4 (*Neisseria* bacteria) to 8 replicates (mock-infected). Red indicates that the signal is higher relative to the rest of the samples while green indicates that the signal is lower. WT: wild type.

Biological processes that were significantly over-represented in host genes regulated specifically in response to live WT *N. lactamica* include cytokine and chemokine mediated signalling pathway at 5 hours, cell proliferation and differentiation at 6 and 7 hours and mRNA transcription and its regulation at 7 hours (Table 5). The expression of genes validated by Q-RT-PCR and involved in these biological processes is shown in a heat map in Figure 8, with their fold changes to live WT *N. lactamica* listed in Tables 6, 7 and 8. Most of the genes (58%) were activated in response to live but not killed *N. lactamica* compared to the mock-infected controls. They were also activated more and for a longer period of time from 5 to 7 hours compared to the response to live WT *N. meningitidis*. Examples of these genes include IL1A and TNF-α, which were involved in the cytokine and chemokine mediated signalling pathway at 5 hours (Table 6), KLF6, ERRFI1 and IL8, which were involved in the cell proliferation and differentiation process at 6 and 7 hours, as well as JUN, which was involved in mRNA transcription and its regulation at 7 hours (Tables 7 and 8).

**Table 5:** Host biological processes specifically associated with live WT *N. lactamica* infection.

	Time points (hour)				
Biological Process	3	4	5	6	7
Cytokine and chemokinemediated signalling pathway	-	-	2.2E-3	-	-
Cell proliferation anddifferentiation	-	-	-	8.5E-3	3.7E-6
mRNA transcription	-	-	-	-	4.8E-3
mRNA transcription regulation	-	-	-	-	2.0E-3

Significance of biological processes is expressed as p-values and dashes indicate no significance was identified at the time point.

**Table 6:** Validated genes regulated specifically in response to live WT *N. lactamica* and associated with cytokine and chemokine mediated signalling pathway at 5 hours.

Human Genbank ID	Symbol	Description	Array	Q-RT-PCR
NM_000575	IL1A	interleukin 1, alpha	3.2	2.0
NM_000594	TNF-α	tumor necrosis factor, alpha	2.2	2.3

**Table 7:** Validated genes regulated specifically in response to live WT *N. lactamica* and associated with cell proliferation and differentiation process at 6 hours.

Human Genbank ID	Symbol	Description	Array	Q-RT-PCR
NM_003670	BHLHB2	basic helix-loop-helix domain containing, class B, 2	2.3	3.1
NM_004354	CCNG2	cyclin G2	3.0	1.7
NM_005228	EGFR	epidermal growth factor receptor	2.1	1.3
NM_001300	KLF6	Kruppel-like factor 6	2.4	2.1
NM_003415	ZNF268	zinc finger protein 268	−2.7	−1.7

**Table 8:** Validated genes regulated specifically in response to live WT *N. lactamica* and associated with mRNA transcription regulation, cell proliferation and differentiation and mRNA transcription processes at 7 hours.

HumanGenbank ID	Symbol	Description	Array	Q-RT-PCR	Biological Processes		
					mRNAtranscriptionregulation	Cellproliferationanddifferentiation	mRNAtranscription
NM_003670	BHLHB2	basic helix-loop-helix domain containing, class B, 2	2.1	2.0	x	x	x
NM_001300	KLF6	Kruppel-like factor 6	2.4	2.3	x	x	x
NM_002894	RBBP8	retinoblastoma binding protein 8	−2.0	−1.6	x	x	x
NM_004234	ZNF235	zinc finger protein 235	−2.0	−2.1	x	x	x
NM_006454	MXD4	MAX dimerization protein 4	−2.0	−1.5	x	x	x
NM_012323	MAFF	v-maf musculoaponeurotic fibrosarcoma oncogene homolog F (avian)	2.0	1.7	x	x	x
NM_002228	JUN	jun oncogene	2.1	1.9	x	x	x
NM_005238	ETS1	v-ets erythroblastosis virus E26 oncogene homolog 1 (avian)	2.3	2.1	x	x	x
NM_021220	OVOL2	ovo-like 2 (Drosophila)	−2.4	−1.9	x		x
NM_015630	EPC2	enhancer of polycomb homolog 2 (Drosophila)	−2.3	−2.1	x		x
NM_006399	BATF	basic leucine zipper transcription factor, ATF-like	−7.7	−2.1	x		x
NM_025209	EPC1	enhancer of polycomb homolog 1 (Drosophila)	−2.1	−1.8	x		x
NM_005902	SMAD3	SMAD family member 3	2.1	1.7	x		x
NM_016270	KLF2	Kruppel-like factor 2 (lung)	2.3	2.0	x		x
NM_020436	SALL4	sal-like 4 (Drosophila)	3.8	2.7	x		x
NM_003111	SP3	Sp3 transcription factor	−2.0	−1.4		x	x
NM_000575	IL1A	interleukin 1, alpha	3.7	2.4		x	
NM_000584	IL8	interleukin 8	2.7	2.1		x	
NM_004354	CCNG2	cyclin G2	2.4	1.8		x	
NM_198267	ING3	inhibitor of growth family, member 3	−2.1	−2.3		x	
NM_018948	ERRFI1	ERBB receptor feedback inhibitor 1	2.1	3.1		x	
NM_032587	CARD6	caspase recruitment domain family, member 6	−2.2	−2.4			x
NM_030665	RAI1	retinoic acid induced 1	2.2	1.6			x

**Figure 9:**
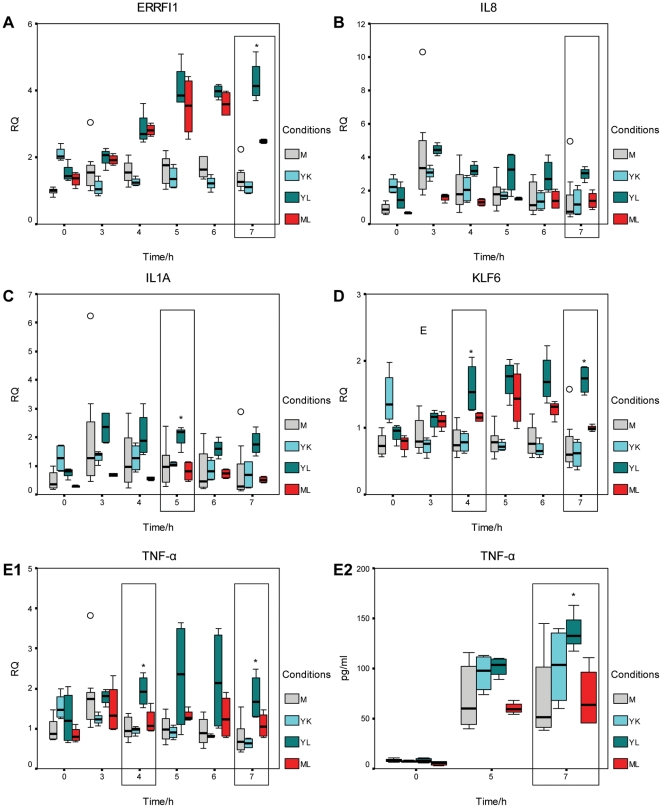
Expression of host genes up-regulated specifically in response to live *N. lactamica*. Activation of ERRFI1, IL8, IL1A, KLF6 and TNF-α was specifically associated with live but not killed *N. lactamica*. This occurred at a time point of 7 hours for ERRFI1 (A) and IL8 (B), at 5 hours for IL1A (C) and at 4 and 7 hours for KLF6 (D) and TNF-α (E1) at the transcript level using Q-RT-PCR. Measurement of TNF-α at the protein level also indicated an up-regulation at the 7 hour time point (E2). Asterisks (*) indicate statistical significance with a p-value of less than 0.05 with respect to mock-infected controls. M: mock-infected, YK: killed WT *N. lactamica*, YL: live WT *N. lactamica*, ML: live WT *N. meningitidis*. RQ: relative quantification. An outlier (0) is defined as between 1.5 to 3× the interquartile range from the 25th or 75th percentile while an extreme data point (E) is defined as more than 3× the interquartile range from the 25th or 75th percentile.

The time course of expression of representative validated genes at the transcript level by Q-RT-PCR and at the protein level by ELISA is shown in Figure 9. The expression of ERRFI1 (a cytoplasmic protein), KLF6 (a transcription factor) and proinflammatory cytokines such as IL1A, IL-8 and TNF-α was higher in *N. lactamica*-infected 16HBE14 epithelial cells compared to those infected with *N. meningitidis*. At the transcript level, this occurred at a time point of 7 hours for ERRFI1 (Figure 9A) and IL8 (Figure 9B), at 5 hours for IL1A (Figure 9C) and at 4 and 7 hours for KLF6 (Figure 9D) and TNF-α (Figure 9E1). Measurement of TNF-α at the protein level also indicated an up-regulation at the 7 hour time point (Figure 9E2). Increased expression of these genes was associated with live but not killed *N. lactamica*.

### Secreted proteins of *N. meningitidis* and *N. lactamica* regulate C1S and TNF-α expression respectively in 16HBE14 cells

Microarray and Q-RT-PCR data indicated a differential expression of host genes involved in immunity and defence in response to *N. meningitidis* and *N. lactamica*. Some of these responses e.g. the down-regulation of C1S in response to *N. meningitidis* and the activation of TNF- α in response to *N. lactamica* were mediated by live bacteria, and in the case of *N. meningitidis* were independent of the presence of capsule or pili, suggesting a mechanism involving active production of mediators. Secreted protein preparations from WT *N. meningitidis* and *N. lactamica* were obtained from supernatants cultured in the exponential phase of growth, treated to remove outer membrane vesicles (by ultracentrifugation), depleted of lipooligosaccharides (by passing through polymyxin B columns) and added to the 16HBE14 cells.

**Figure 10:**
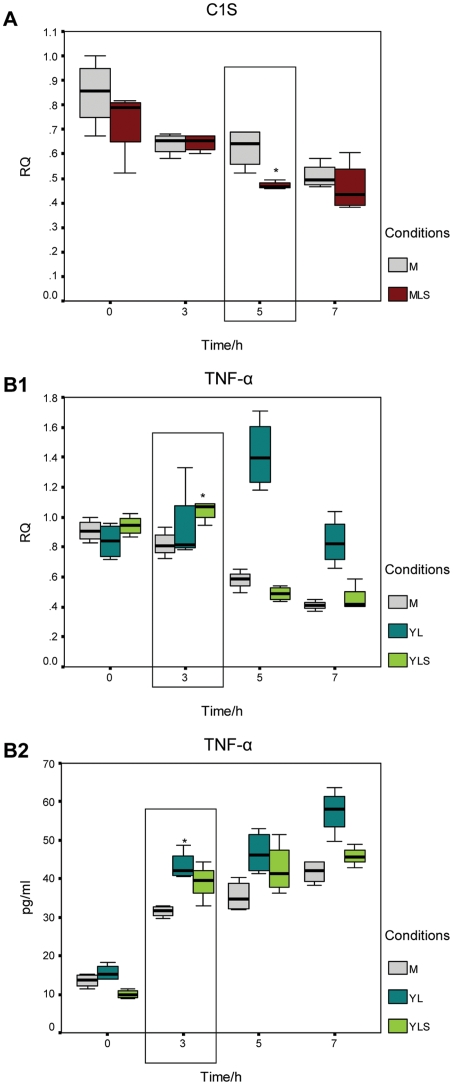
Secreted proteins of *N. meningitidis* and *N. lactamica* regulate C1S and TNF-α expression, respectively. Treatment of host epithelial cells with secreted protein preparations from WT *N. meningitidis* indicate that secreted proteins may be involved in the down-regulation of C1S at 5 hours at the transcript level (A). In contrast, secreted protein preparations from WT *N. lactamica* resulted in an activation of TNF-α at 3 hours at both the transcript (B1) and the protein level (B2). Asterisks (*) indicate statistical significance with a p-value of less than 0.05 with respect to mock-infected controls. M: mock-infected, MLS: WT *N. meningitidis* secreted protein preparations, YL: live WT *N. lactamica*, YLS: WT *N. lactamica* secreted protein preparations. RQ: relative quantification.

*N. meningitidis* and *N. lactamica* secreted protein preparations contained less than 0.1 endotoxin units per ml as determined by the Limulus amoebocyte lysate (LAL) assay. Contamination of secreted protein preparations by outer membrane proteins and/or outer membrane vesicles was assessed by the presence or absence of NspA by Western blotting. NspA is an outer membrane protein present in both *N. meningitidis* and *N. lactamica* and is a major component of their outer membrane vesicles [30]. No NspA was detected in either the *N. meningitidis* and *N. lactamica* secreted protein preparations (data not shown). The addition of *N. meningitidis* and *N. lactamica* secreted protein preparations resulted in the down-regulation of C1S at 5 hours at the transcript level (Figure 10A) and the activation of TNF-α at 3 hours at both the transcript and protein levels (Figure 10 B1 and B2), respectively.

## Discussion

### The influence of association and invasion of *N. lactamica* and *N. meningitidis* on gene expression in 16HBE14 epithelial cells

This is the first study to investigate the comparative host response of human respiratory tract cells to *N. lactamica* and *N. meningitidis* using a whole genome microarray platform. Emphasis has been placed on early events (0–7 hours). Firstly, the extent of association and invasion of the two bacteria with 16HBE14 bronchial epithelial cells were compared. There were no significant differences in association and invasion of *N. lactamica* or *N. meningitidis* at 3 hours. We have shown that the early suppression of host defence genes is specifically associated with live *N. meningitidis* 3 hours post infection, which suggests that this specific host response is due to bacterial differences rather than their numbers per se. At later time points of 5 and 7 hours however, more *N. lactamica* associated and invaded the 16HBE14 cells, compared to *N. meningitidis*. There was a concomitant increase in activation of proinflammatory processes associated with *N. lactamica* at these later time points and this may reflect the greater numbers of bacteria present. Another strain of *N. lactamica* (NL4.1) has also been recently shown to invade epithelial cells derived from the respiratory tract [22].

In this study, compared to WT *N. meningitidis*, the *cap*- and *pilE*- mutants associated and invaded 16HBE14 cells to greater and lesser extents, respectively. These findings are consistent with previous studies. For example, it is known that the expression of the capsular polysaccharide inhibits the invasion of the nasopharyngeal barrier by masking the meningococcal adhesins and invasins [11]. In addition, a role for *N. meningitidis* pili in adherence to epithelial cells has been well documented [6], [12].

### Utilisation of host energy production processes by colonising *Neisseria*

An up-regulation of host genes involved in the biological processes of phosphate metabolism and glycolysis was associated with both live *N. lactamica* and *N. meningitidis*. This suggests that the two bacteria share a common mechanism for successful colonisation whereby they both actively utilise host resources as a way to survive and adapt in the host. The genes STC1 and STC2 (from the regulation of phosphate metabolism pathway) encode members of a family of secreted homodimeric glycoproteins which are involved in phosphate transport, cell metabolism, and cellular calcium/phosphate homeostasis. ENO2 (enolase 2), HK2 (hexokinase 2), PFKFB3 and PFKFB4 (phosphofructokinases 3 and 4) are genes involved in carbohydrate metabolism including glycolysis.

The clustering of host genes regulated in response to both bacteria into the categories of regulation of phosphate metabolism and carbohydrate metabolism suggests that the human host cell represents a milieu rich in nutrients for bacterial growth and a source of energy. Grifantini et al. [23] observed that cell contact of *N. lactamica* and *N. meningitidis* with 16HBE14 cells resulted in the down-regulation of several bacterial genes involved in metabolism. To explain this, the authors suggested that the bacteria were able to utilise part of the ATP synthesised by the host. Similarly, when Dietrich et al. [31] analysed the transcriptome of *N. meningitidis* after contact with epithelial cells (Hela cells) and human brain microvascular endothelial cells, a high proportion of the differentially regulated genes were involved in central metabolism. In addition, genes involved in cell metabolism, particularly in energy production, were found to have increased transcription in human brain endothelial cells in response to *N. meningitidis* [17].

The *pfk* gene (encoding phosphofructokinase) is not found in the genomes of *N. meningitidis* and *N. lactamica*, which explains the lack of a functional glycolytic pathway in these bacteria [26], [32], [33]. Under anaerobic conditions, where oxidative phosphorylation cannot occur, *pfk* is essential as the glycolytic process is important for the production of energy. For *Neisseria* however, which colonises the aerobic nasopharyngeal mucosa, sufficient energy can be liberated from the substrate by oxidative phosphorylation, and the glycolytic process (in which phosphofructokinase is an enzyme) is not essential. Although *Neisseria* does not carry out glycolysis, host glycolytic enzymes (ENO2, HK2, PFKFB3 and PFKFB4) were up-regulated in response to the bacteria in this study. It is tempting to speculate that the bacteria may be responsible for this up-regulation so as to increase the amount of ATP produced by the host, which in turn may be available for use by the bacteria. Our results are broadly in agreement with another study describing a commensal bacterium using a similar strategy to survive in the human host. *Bacteroides thetaiotaomicron*, a component of the intestinal microflora of humans uses epithelial fucosylated glycans as a source of energy in the highly competitive intestinal ecosystem. In this way, the host appears to be a participant in providing for the nutritional needs of the bacteria [34].

### *N. meningitidis* down-regulates immune response genes while *N. lactamica* initiates a proinflammatory response

Results from the microarray validated by Q-RT-PCR, and in selected cases at the protein level, indicate a down-regulation of genes such as C1S, LCN2 and PI3 after infection with *N. meningitidis* relative to *N. lactamica*. In contrast, there was a greater activation of genes such as TNF-α, IL1A, IL8, JUN, ERRFI1 and KLF6 after infection with *N. lactamica* relative to *N. meningitidis*. The results suggest that, in broad terms, *N. meningitidis* is associated with a suppression of the host defence response while *N. lactamica* is associated with an activation of the proinflammatory response.

Besides our study, there have been very few investigations comparing host responses to *N. meningitidis* and *N. lactamica*. In one such study looking at a range of limited cytokine and chemokine responses in human meningioma cells, *N. meningitidis* induced higher amounts of proinflammatory markers such as IL8 compared to *N. lactamica* at much later time points of up to 48 hours [35]. It is well documented that during invasive meningococcal disease, potentially life-threatening meningitis and septicaemia arises from the host due to the overwhelming amount of proinflammatory cytokines and chemokines produced [36], and thus it is not surprising host cells derived from the blood and the central nervous system respond analogously *in vitro*. In contrast to this study, we used an epithelial cell line from the respiratory tract and followed responses at early time points of up to 7 hours. Many factors will determine the relative host gene expression in response to *N. meningitidis* and *N. lactamica* and these include cell lineage and the time after infection sampled.

In this study, a specific down-regulation of host defence genes (C1S, LCN2 and PI3) was associated with live *N. meningitidis*. C1s encodes a serine protease, which is a major constituent of the human complement subcomponent C1. C1s associates with two other complement components C1r and C1q in order to yield the first component of the serum complement system. It is widely known that an effective complement system is pivotal for host resistance against *N. meningitidis*. It is, therefore, not surprising that *N. meningitidis* has been found to exploit two negative complement regulators from its human host, factor H [37] and C4 binding protein [9] to reduce the effectiveness of the host complement defence system. For example, *N. meningitidis* mimics the mechanism by which host cells regulate complement activation on their surface by facilitating the high affinity interaction between factor H and factor H binding protein on the bacterium. This suggests that *N. meningitidis* could rapidly sequester factor H, an alternative pathway inhibitor, and avoid clearance by the complement system [38]. Suppression of C1S, another component of the classical complement pathway, (see Figure 7A and Figure 10A), could be another mechanism to subvert host defences during colonisation.

LCN2 and PI3 encode antimicrobial peptides. LCN2 inhibits microbial growth by limiting iron availability [39] while PI3 is a low molecular weight cationic peptide and acts as an antimicrobial defensin-like molecule with the ability to eliminate pulmonary pathogens [40]. The expression and secretion of PI3 is induced in human keratinocytes [41] and in bronchial epithelial cells [42] after exposure to *Pseudomonas aeruginosa*. Other pathogens e.g. adenoviruses have been found to be responsible for the suppression of PI3 in primary human bronchial epithelial cells [43]. In addition, another antimicrobial peptide LL-37 has also been found to be consistently suppressed by *Neisseria gonorrhoeae* (another pathogenic species of *Neisseria*) in a cervical epithelial cell line [44]. Down-regulation of antimicrobial peptides like LCN2 and PI3 by *N. meningitidis* may be a mechanism to reduce the ability of the host to clear the bacterium and to promote colonisation.

The expression of C1S, LCN2 and PI3 is not only specifically down-regulated in response to live WT *N. meningitidis*, but also in response to the *N. meningitidis cap*- and *pilE*- mutants, despite their different extent of association and invasion of host epithelial cells. This suggested an active bacterial process involved in the down-regulation of these genes. Co-incubation of 16HBE14 cells with preparations of WT *N. meningitidis* secreted proteins indicated their involvement in the down-regulation of C1S. However, they had no effect on the expression of LCN2 and PI3, indicating that other extracellular bacterial components, such as outer membrane vesicles (absent in our secreted protein preparations) may have a role in the differential expression of LCN2 and PI3. Other alternative explanations are that the meningococcal secreted protein(s) mediating differential gene expression of LCN2 and PI3 is produced by bacteria growing in the presence of 16HBE14 cells/serum (rather than defined medium) or when *N. meningitidis* is attached to or has invaded host cells. *N. meningitidis* and *N. lactamica* were grown to log phase in defined medium to allow pure bacterial protein secreted preparations. We did consider the use of supernatants obtained from co-cultures of bacteria with 16HBE14 cells. However, it would have been less clear as to whether any changes in host gene expression were due to bacterial or host derived factors, and thus, this approach was not used.

It has been shown by Liu et al. [21] in another human bronchial epithelial cell line (BEAS-2B) that *N. lactamica* PorB binds to TLR2 and is a poorer inducer of proinflammatory mediators compared to that from *N. meningitidis*. Tezera et al. [22] reported that *N. lactamica* NL4.1 protects mucosal barrier integrity by suppressing *N. meningitidis*-induced inflammation by increasing the expression of PPARγ and inhibiting NFκβ activity. Secretory TNF-α and IL-8 were found to be elevated after challenge with *N. meningitidis* compared to *N. lactamica* at 24 hours post infection. In our study, however, relative to *N. meningitidis*, TNF-α and IL-8 expression was up-regulated in response to *N. lactamica* at time points up to 7 hours post infection. Although our results appear to contrast with these two studies, it may not be reasonable to compare our findings with theirs, as our experimental set ups were different in terms of cell line, infection time points, bacterial strains and multiplicity of infection used. With 16HBE14 cells, proinflammatory molecule expression was inhibited by the bacterial pathogen *Yersinia pseudotuberculosis* [45]. In addition, our array data indicates that the expression of TLR2 at the transcript level could not be detected in our cell line and both PPARγ and NFκβ were not significantly differentially expressed in response to *N. meningitidis* or to *N. lactamica*, compared to mock-infected controls, at early time points from 3 to 7 hours post infection.

However, our results are not mutually incompatible with those from other studies. Our study found that whilst our *N. lactamica* strain can induce the expression of proinflammatory markers such as TNF-α, IL1A, IL-8 and JUN, it is also able to activate negative regulators of inflammation, preventing an excessive proinflammatory response within the respiratory tract, which may disrupt epithelial barriers [46]. Two up-regulated genes were found in this study (in addition to TNF-α, IL1A, IL-8 and JUN) that may play a role to prevent excessive production of proinflammatory cytokines and over activation of the epithelial cell barrier. One of them is ERRFI1, a cytoplasmic protein whose expression is up-regulated with cell growth [47]. It shares significant homology with the protein product of rat gene-33, which is induced during cell stress and mediates cell signalling [48], [49]. Activation of epidermal growth factor receptor has been shown to contribute to the proinflammatory response (by the release of IL-8) in respiratory tract epithelial cells [50] and ERRFI1 has recently been suggested to act as a negative feedback inhibitor of epidermal growth factor receptor signalling through a direct, physical interaction with the epidermal growth factor receptor [51]. The other gene is KLF6, which belongs to the Kruppel-like protein family and inhibits the activity of JUN. Together with KLF2, they have an important regulatory role in controlling and inhibiting numerous host cellular processes, including phagocytosis, proinflammatory cytokine expression and cell proliferation [52].

Certain experimental models investigating host responses to respiratory pathogens have attempted to mimic the cooling effect of constant evaporation from respiratory mucosal surfaces. However, we chose, in line with other previous studies [53], [54], to investigate airway epithelial colonisation by meningococci at 37°C. *N. meningitidis* strains grown on solid agar at 30°C and 37°C were described as having piliated and non-piliated phenotypes, respectively [55]. *N. meningitidis* MC58 was not used in the latter study. Whilst we did not quantify either pilin-related gene or protein expression, we observed a clear difference in association and invasion of WT *N. meningitidis* MC58 compared to the *pilE*- mutant. This suggests that in our study (at 37°C), WT *N. meningitidis* MC58 had a functional piliated phenotype.

Another aspect to consider is that differences in the numbers of adherent and invading *N. meningitidis* and *N. lactamica* per se might contribute to the host gene and/or protein expression changes observed. For example, the comparative higher activation of proinflammatory genes like TNF-α and IL8 by *N. lactamica*, compared to *N. meningitidis*, may reflect the greater numbers of *N. lactamica* associating and invading the host cells.

In our model both *N. meningitidis* and *N. lactamica* adhered to and invaded 16HBE14 cells. Whether bacteria that are adherent induce host gene responses that are different to those that have invaded is unknown but may contribute to the results obtained. One possibility, given that *N. meningitidis* has the potential to cause invasive disease while *N. lactamica* does not, is that host responses of respiratory epithelial cells induced by invasive bacteria may differ more between the two organisms than to extracellular colonisation.

### Model of early colonisation by *N. meningitidis and N. lactamica*

**Figure 11:**
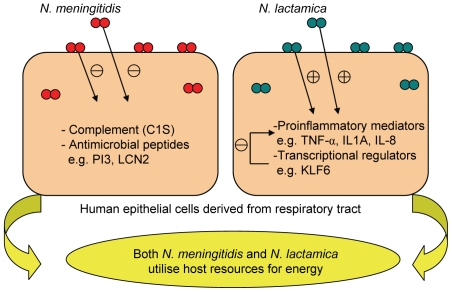
Postulated model of early colonisation by *N. meningitidis* and *N. lactamica*. Host metabolic and energy production processes were associated with both neisserial species, suggesting that *N. meningitidis* and *N. lactamica* utilise host resources for energy. In addition, differential host responses to *N. meningitidis* and *N. lactamica* may indicate different colonisation processes. *N. meningitidis* down-regulates host defence genes such as complement (C1S) and antimicrobial peptides (for example PI3 and LCN2), reducing the ability of the host to clear the bacterium and thus promote colonisation. This may contribute to the ability of *N. meningitidis* to cause invasive disease in an environment where host defences are compromised. In contrast, *N. lactamica* does not evade the host immune response as seen from the activation of genes such as TNF-α, IL1A and IL-8, and that the expression of these cytokines may alert the host to its presence and potentially prevent or limit the bacterium from entering the bloodstream and causing invasive disease. Despite this proinflammatory response, *N. lactamica* continues to colonise the nasopharynx. This is likely to be due to an up-regulation of genes such as KLF6 that regulate and prevent an over activation of the proinflammatory response so that the commensal is not cleared completely by the host.

Based on our results, we propose that *N. meningitidis* and *N. lactamica* use both shared and different mechanisms to colonise host respiratory epithelial cells (Figure 11). Both bacteria appear to use similar strategies to utilise host energy resources for growth. However, *N. meningitidis* appears to down-regulate host defence genes such as those encoding antimicrobial peptides such as PI3 and LCN2 and complement components such as C1S, as a strategy to maintain colonisation. This may also explain why *N. meningitidis* has the potential to cause invasive disease in an environment where host defences are compromised. In contrast, *N. lactamica* does not evade the host immune response, as indicated by activation of genes such as TNF-α, IL1A and IL-8, and that the expression of these cytokines may alert the host to its presence and potentially prevent or limit the bacterium from entering the blood and causing invasive disease. Despite this proinflammatory response, *N. lactamica* continues to colonise the nasopharynx. This is likely to be due to an up-regulation of genes such as KLF6 that regulate and prevent an over activation of the proinflammatory response so that the commensal is not cleared completely by the host.

Neisserial secreted proteins appeared to be responsible for some of the host responses specific to *N. meningitidis* or *N. lactamica*. Bioinformatic and proteomic studies have identified proteins that are known or predicted to be secreted by *N. meningitidis* and *N. lactamica*. A review by van Ulsen and Tommassen [56] used the available genomes of *N. meningitidis* and of *N. lactamica* (ST640) to identify genes encoding predicted secreted proteins specific to each bacterium. Our results suggest that secreted proteins can regulate gene expression in respiratory epithelial cells.

In conclusion, our results show that whilst both *N. lactamica* and *N. meningitidis* colonise respiratory tract epithelial cells, they have both common and distinct effects on host gene expression and these may be associated with their respective ability to colonise or cause disease.

## Materials and Methods

### Bacteria and growth conditions

*N. lactamica* Y92-1009 was obtained from the Health Protection Agency (Porton Down, UK). WT *N. meningitidis* MC58 and its capsule (*cap*-) and pilus (*pilE*-) mutants have been described previously [12], [57], [58]. Formaldehyde inactivation of WT *N. meningitidis* MC58 and *N. lactamica* Y92-1009 was carried out as described previously [59]. *Neisseria* were routinely propagated on gonococcal agar supplemented with 1% Vitox (sGC) or on brain heart infusion agar (BHI) (BD Diagnostics, USA) at 37°C in 5% CO_2_. Antibiotics were used at the following concentrations for selective growth of the *N. meningitidis cap*- and *pilE*- mutants: kanamycin 150 µg/ml and erythromycin 5 µg/ml.

### Growth of 16HBE14 bronchial epithelial cells

The 16HBE14 bronchial epithelial cell line is derived from primary human bronchial epithelial cells and transformed by SV40 large T antigen [60]. It has been shown to retain differentiated epithelial morphology and functions and has also been used extensively in cystic fibrosis research [61]. 16HBE14 cells were cultured in Dulbecco's Modified Eagle Medium (DMEM) supplemented with 2 mM L-glutamine, 1% penicillin and streptomycin and 10% heat inactivated foetal bovine serum (HIFBS) in a humidified incubator at 37°C with 5% CO_2_. Growth media, antibiotics, supplements and foetal bovine serum were obtained from Invitrogen.

### Epithelial association and invasion assays

16HBE14 cells were seeded in 24-well plates (Nunc, Thermo Fisher Scientific, USA) and grown to a confluent monolayer (approximately 2×10^5^ cells per well) in DMEM supplemented with 2 mM L-glutamine and 10% HIFBS at 37°C in 5% CO_2_. Bacteria were added at a multiplicity of infection of 30 (6×10^6^ cfu per well) for both association and invasion assays which were performed as previously described [62]. At time points of 3, 5 and 7 hours, wells were incubated with 1% saponin for 10 minutes at 37°C and appropriate dilutions (in DMEM) plated out to obtain viable counts.

### Treatment of 16HBE14 human epithelial cells with N. meningitidis, N. lactamica or neisserial secreted protein preparations

16HBE14 cells were seeded into 6-well plates and incubated in DMEM supplemented with 2 mM L-glutamine and 10% HIFBS at 37°C with 5% CO_2_. Confluent epithelial cells (approximately 9.5×10^5^ cells per well) were washed 3 times with phosphate buffered saline (PBS) and its medium changed before infection with 1.3×10^7^ cfu per well of *N. meningitidis* or *N. lactamica* (washed with DMEM prior to infection) or treatment with neisserial secreted protein preparations (2 µg per well). At various time points from 0 to 7 hours, the epithelial host cells were washed with PBS and harvested with Trizol (Sigma, UK). For each condition, there were 4 to 8 biological replicates. For neisserial infection experiments, initial bacterial inocula were determined by plating out on sGC or BHI agar for enumeration of viable organisms.

### RNA extraction and quantification

Total cellular RNA from human epithelial cells was extracted using the Qiagen RNeasy Mini Kit using on column DNase 1 treatment according to manufacturer's instructions (Qiagen, USA). Quantification of RNA samples was performed by checking the absorbance at 260 nm using a NanoDrop 1000 instrument (NanoDrop Technologies, Wilmington, DE).

### RNA amplification and microarray hybridisation

Whole genome microarray hybridisations were performed as described previously [63] on RNA samples from mock-infected host cells or those co-cultured with *Neisseria*. Briefly, total RNA (500 ng) was amplified in a single-round of in vitro transcription amplification that allowed incorporation of biotin-labelled nucleotides using the Illumina TotalPrep RNA Amplification Kit (Ambion, USA) according to the manufacturer's instructions. Output cRNA was quantified using the NanoDrop ND-1000 UV-Vis Spectrophotometer. cRNA (750 ng) of each sample was hybridised to an Illumina HumanRef-8 V2 BeadChip (containing probes to 20,589 RefSeq gene sequences) at 58°C for 18 hours following the manufacturer's instructions (Illumina, USA). This was followed by washing, blocking, and streptavidin-Cy3 staining steps, followed by scanning with a high resolution Illumina Bead Array Reader confocal scanner, all carried out following manufacturer's instructions.

### Microarray data analysis

The scanned microarray images were analysed, data extracted and background subtracted using the Illumina Bead Studio v3.1 software (Illumina, USA). The data exported from Bead Studio was then further analysed using Genespring GX7.3 software (Agilent Technologies, USA). Data transformation was corrected for low signals, setting measurement less than 0.01 to 0.01. The standard normalisation procedures were performed as recommended by the Genespring software for one colour array. Per-chip normalisation, where each measurement was divided by the 50th percentile of all measurements in that sample, and per-gene normalisation to median, where each gene was divided by the median of its measurements in all samples, were done. Genes that have a less than 99% confidence of detection above background levels in all the arrays were excluded from the final analysis, leaving 13,226 genes available for statistical differential expression analysis. The normalised signal of each condition was the geometric mean of the normalised signals of its individual biological replicates. Fully annotated microarray data have been deposited in Gene Expression Omnibus (GEO) under accession number GSE27557 (http://www.ncbi.nlm.nih.gov/geo/query/acc.cgi?token=jdublkkyqamgyzc&acc=GSE27557).

Group comparisons were made between mock-infected cells and those challenged with bacteria or neisserial secreted protein preparations over a 7 hour time course. Differentially expressed genes were selected from the normalised data using the program Significance Analysis of Microarrays [64], which was incorporated into Genespring GX 7.3. For all comparisons, a false discovery rate of 5% was used. In addition, to remove low signal genes that could give false positive results due to a lack of sensitivity, significant genes, which did not have a raw intensity value of more than 50 in at least half of the samples in the smaller group of comparison, were discounted. For comparisons between mock-infected cells and cells challenged with (live or killed) WT bacteria, significant genes with a less than 2-fold change difference were removed to increase stringency.

Functional clustering of differentially expressed genes was assessed using the Applied Biosystems online program “Panther” gene expression analysis system [29]. These genes were compared with those in the Illumina array reference list to statistically determine over representation of functional categories. A Bonferroni corrected p-value of less than 0.05 was considered significant.

Heat maps were generated using log_2_ transformed normalised values. The genes were further clustered in Gene Cluster 3.0 (Stanford University, USA) with uncentered correlation as the similarity metric and average linkage as the clustering method. The output file was then visualised using TreeView (EisenSoftware, USA). Red indicates that the gene's signal is higher relative to the rest of the samples while green indicates that the gene's signal is lower relative to the rest of the samples.

### Q-RT-PCR

TaqMan Low Density Array cards were designed to validate a subset of genes that were differentially expressed in the array experiments (Applied Biosystems, USA). Total RNA (0.5 µg) was converted to cDNA using a High-Capacity cDNA archive kit (Applied Biosystems, USA). The Q-RT-PCR reactions were run on an ABI 7900 system (Applied Biosystems, USA). Data was analysed using the SDS2.2 software where baseline and threshold settings were automatically adjusted. Relative gene expression levels were obtained using the 2^−ΔΔC^_T_ method [65] with the 18S housekeeping gene used for normalisation. In brief, this method uses a single sample, termed the “calibrator sample,” as a comparator for every unknown sample's gene expression level. In this case, we chose a mock-infected sample at 0 hour time point to be the calibrator sample. The calibrator is analysed on every assay plate with the unknown samples of interest. The relative quantification (RQ) value is calculated using the following formula: RQ = 2^−ΔΔC^_T_, where ΔΔC_T_ = (C_T_ of gene of interest in unknown sample -C_T_ of 18S gene in unknown sample) - (C_T_ of gene of interest in calibrator -C_T_ of 18S gene in calibrator). Box plots indicate the median RQ value within a group of biological replicates.

The response of human epithelial cell genes to secreted neisserial proteins were determined by Q-RT-PCR using the Fluidigm chip technology (Fluidigm, USA). Briefly, 0.5 µg total RNA was converted to cDNA using the High-Capacity cDNA archive kit. From this, the cDNA was preamplified using the TaqMan PreAmp master mix (Applied Biosystems, USA). The cDNA, TaqMan universal PCR master mix, DA sample loading reagent, TaqMan gene primers and DA assay loading reagent were mixed accordingly and loaded into a specialised integrated fluidic circuit controller following manufacturer's instructions. The Q-RT-PCR reactions were read using the Biomark data collection machine (Fluidigm, USA). Data was analysed using the Biomark Real-Time PCR analysis software (Fluidigm, USA). Relative gene expression levels were obtained after normalisation to 18S rRNA. Relative quantification levels are obtained with respect to mock-infected sample at 0 hour time point.

### ELISAs

After treatment of cells with either whole bacteria or bacterial secreted proteins, supernatants were harvested, centrifuged for 10 minutes at 3220 g and passed through 0.2 µm filters to remove the bacteria. PI3 and TNF-α measurements were performed according to instructions in the ELISA kits (R&D systems and Bio-Rad, USA).

### Neisserial secreted proteins

*N. meningitidis* and *N. lactamica* were grown in RPMI media (Sigma, UK) supplemented with amino acids when necessary [66]. Crude neisserial secreted proteins were harvested as described in Robinson et al. [20] with some modifications. Briefly, the bacteria were grown at 37°C with shaking at 200 rpm. Bacterial supernatants were harvested at log phase and ultracentrifuged at 40,000 g for 1 hour. The sample was then concentrated 20 times with a 10 kDa molecular weight cut off using Vivaspin ultrafiltration spin columns (Sartorius Stedim, Germany). Endotoxin removal was carried out using the Detoxi-Gel Endotoxin Removing Gel consisting of immobilised polymyxin B (Pierce, Thermo Fisher Scientific, USA) until there was less than 0.1 EU/ml, as measured by LAL assay (Lonza,USA).

### Statistical analysis

For the association and invasion assays, as well as the microarray validation by Q-RT-PCR, mean values were used. Significance of difference was determined using the Student's T-test (assuming unequal variance), whereby p-values of less than 0.05 were considered to represent significance.

The expression of selected genes that were studied in detail over a time course at the transcript and protein level were analysed by SPSS11 (IBM, USA) software. Box plots representing the interquartile range, the median and the highest and lowest values among the biological replicates were drawn. The Mann-Whitney test was used to compare between conditions to determine if the expression of a gene was significantly different (with a p-value of less than 0.05).

## Supporting Information

Table S1 Microarray and Q-RT-PCR results showing host gene expression fold changes from 5 to 7 hours in response to live WT *N. lactamica* and *N. meningitidis*.
(DOC)

## References

[1] Serruto D, Adu-Bobie J, Capecchi B, Rappuoli R, Pizza M (2004). Biotechnology and vaccines: application of functional genomics to *Neisseria meningitidis* and other bacterial pathogens.. J Biotechnol.

[2] Gold R, Goldschneider I, Lepow ML, Draper TF, Randolph M (1978). Carriage of *Neisseria meningitidis* and *Neisseria lactamica* in infants and children.. J Infect Dis.

[3] Olsen SF, Djurhuus B, Rasmussen K, Joensen HD, Larsen SO (1991). Pharyngeal carriage of *Neisseria meningitidis* and *Neisseria lactamica* in households with infants within areas with high and low incidences of meningococcal disease.. Epidemiol Infect.

[4] Caugant DA, Maiden MC (2009). Meningococcal carriage and disease–population biology and evolution.. Vaccine.

[5] Evans CM, Pratt CB, Matheson M, Vaughan TE, Findlow J (2011). Nasopharyngeal colonization by *Neisseria lactamica* and induction of protective immunity against *Neisseria meningitidis*.. Clin Infect Dis.

[6] Pujol C, Eugene E, de Saint Martin L, Nassif X (1997). Interaction of *Neisseria meningitidis* with a polarized monolayer of epithelial cells.. Infect Immun.

[7] Bennett JS, Bentley SD, Vernikos GS, Quail MA, Cherevach I (2010). Independent evolution of the core and accessory gene sets in the genus *Neisseria*: insights gained from the genome of *Neisseria lactamica* isolate 020-06.. BMC Genomics.

[8] Read RC, Zimmerli S, Broaddus C, Sanan DA, Stephens DS (1996). The (alpha2–>8)-linked polysialic acid capsule of group B *Neisseria meningitidis* modifies multiple steps during interaction with human macrophages.. Infect Immun.

[9] Schneider MC, Exley RM, Ram S, Sim RB, Tang CM (2007). Interactions between *Neisseria meningitidis* and the complement system.. Trends Microbiol.

[10] Virji M (1996). Meningococcal disease: epidemiology and pathogenesis.. Trends Microbiol.

[11] Hammerschmidt S, Hilse R, van Putten JP, Gerardy-Schahn R, Unkmeir A (1996). Modulation of cell surface sialic acid expression in *Neisseria meningitidis* via a transposable genetic element.. Embo J.

[12] Virji M, Saunders JR, Sims G, Makepeace K, Maskell D (1993). Pilus-facilitated adherence of *Neisseria meningitidis* to human epithelial and endothelial cells: modulation of adherence phenotype occurs concurrently with changes in primary amino acid sequence and the glycosylation status of pilin.. Mol Microbiol.

[13] Schubert-Unkmeir A, Slanina H, Frosch M (2009). Mammalian cell transcriptome in response to meningitis-causing pathogens.. Expert Rev Mol Diagn.

[14] Bonnah RA, Muckenthaler MU, Carlson H, Minana B, Enns CA (2004). Expression of epithelial cell iron-related genes upon infection by *Neisseria meningitidis*.. Cell Microbiol.

[15] Plant L, Asp V, Lovkvist L, Sundqvist J, Jonsson AB (2004). Epithelial cell responses induced upon adherence of pathogenic *Neisseria*.. Cell Microbiol.

[16] Linhartova I, Basler M, Ichikawa J, Pelicic V, Osicka R (2006). Meningococcal adhesion suppresses proapoptotic gene expression and promotes expression of genes supporting early embryonic and cytoprotective signaling of human endothelial cells.. FEMS Microbiol Lett.

[17] Schubert-Unkmeir A, Sokolova O, Panzner U, Eigenthaler M, Frosch M (2007). Gene expression pattern in human brain endothelial cells in response to *Neisseria meningitidis*.. Infect Immun.

[18] Pathan N, Hemingway CA, Alizadeh AA, Stephens AC, Boldrick JC (2004). Role of interleukin 6 in myocardial dysfunction of meningococcal septic shock.. Lancet.

[19] Wells DB, Tighe PJ, Wooldridge KG, Robinson K, Ala' Aldeen DA (2001). Differential gene expression during meningeal-meningococcal interaction: evidence for self-defense and early release of cytokines and chemokines.. Infect Immun.

[20] Robinson K, Taraktsoglou M, Rowe KS, Wooldridge KG, Ala'Aldeen DA (2004). Secreted proteins from *Neisseria meningitidis* mediate differential human gene expression and immune activation.. Cell Microbiol.

[21] Liu X, Wetzler LM, Nascimento LO, Massari P (2010). Human airway epithelial cell responses to *Neisseria lactamica* and purified porin via Toll-like receptor 2-dependent signaling.. Infect Immun.

[22] Tezera L, Hampton J, Jackson S, Davenport V (2010). *Neisseria lactamica* attenuates TLR 1/2 - induced cytokine responses in nasopharyngeal epithelial cells using PPAR-gamma.. Cell Microbiol.

[23] Grifantini R, Bartolini E, Muzzi A, Draghi M, Frigimelica E (2002). Gene expression profile in *Neisseria meningitidis* and *Neisseria lactamica* upon host-cell contact: from basic research to vaccine development.. Ann N Y Acad Sci.

[24] Grifantini R, Bartolini E, Muzzi A, Draghi M, Frigimelica E (2002). Previously unrecognized vaccine candidates against group B meningococcus identified by DNA microarrays.. Nat Biotechnol.

[25] Chamot-Rooke J, Mikaty G, Malosse C, Soyer M, Dumont A (2011). Posttranslational modification of pili upon cell contact triggers *N. meningitidis* dissemination.. Science.

[26] Tettelin H, Saunders NJ, Heidelberg J, Jeffries AC, Nelson KE (2000). Complete genome sequence of *Neisseria meningitidis* serogroup B strain MC58.. Science.

[27] Gorringe AR, Taylor S, Brookes C, Matheson M, Finney M (2009). Phase I safety and immunogenicity study of a candidate meningococcal disease vaccine based on *Neisseria lactamica* outer membrane vesicles.. Clin Vaccine Immunol.

[28] Vaughan TE, Skipp PJ, O'Connor CD, Hudson MJ, Vipond R (2006). Proteomic analysis of *Neisseria lactamica* and *Neisseria meningitidis* outer membrane vesicle vaccine antigens.. Vaccine.

[29] Thomas PD, Kejariwal A, Guo N, Mi H, Campbell MJ (2006). Applications for protein sequence-function evolution data: mRNA/protein expression analysis and coding SNP scoring tools.. Nucleic Acids Res.

[30] Mukhopadhyay TK, Halliwell D, O'Dwyer C, Shamlou PA, Levy MS (2005). Rapid characterization of outer-membrane proteins in *Neisseria lactamica* by SELDI-TOF-MS (surface-enhanced laser desorption ionization-time-of-flight MS) for use in a meningococcal vaccine.. Biotechnol Appl Biochem.

[31] Dietrich G, Kurz S, Hubner C, Aepinus C, Theiss S (2003). Transcriptome analysis of *Neisseria meningitidis* during infection.. J Bacteriol.

[32] Baart GJ, Langenhof M, van de Waterbeemd B, Hamstra HJ, Zomer B (2010). Expression of phosphofructokinase in *Neisseria meningitidis*.. Microbiology.

[33] McNeil LK, Reich C, Aziz RK, Bartels D, Cohoon M (2007). The National Microbial Pathogen Database Resource (NMPDR): a genomics platform based on subsystem annotation.. Nucleic Acids Res.

[34] Hooper LV, Xu J, Falk PG, Midtvedt T, Gordon JI (1999). A molecular sensor that allows a gut commensal to control its nutrient foundation in a competitive ecosystem.. Proc Natl Acad Sci U S A.

[35] Fowler MI, Yin KY, Humphries HE, Heckels JE, Christodoulides M (2006). Comparison of the inflammatory responses of human meningeal cells following challenge with *Neisseria lactamica* and with *Neisseria meningitidis*.. Infect Immun.

[36] van Deuren M, Brandtzaeg P, van der Meer JW (2000). Update on meningococcal disease with emphasis on pathogenesis and clinical management.. Clin Microbiol Rev.

[37] Schneider MC, Exley RM, Chan H, Feavers I, Kang YH (2006). Functional significance of factor H binding to *Neisseria meningitidis*.. J Immunol.

[38] Schneider MC, Prosser BE, Caesar JJ, Kugelberg E, Li S (2009). *Neisseria meningitidis* recruits factor H using protein mimicry of host carbohydrates.. Nature.

[39] Reigstad CS, Hultgren SJ, Gordon JI (2007). Functional genomic studies of uropathogenic *Escherichia coli* and host urothelial cells when intracellular bacterial communities are assembled.. J Biol Chem.

[40] Simpson AJ, Maxwell AI, Govan JR, Haslett C, Sallenave JM (1999). Elafin (elastase-specific inhibitor) has anti-microbial activity against gram-positive and gram-negative respiratory pathogens.. FEBS Lett.

[41] Meyer-Hoffert U, Wichmann N, Schwichtenberg L, White PC, Wiedow O (2003). Supernatants of *Pseudomonas aeruginosa* induce the *Pseudomonas*-specific antibiotic elafin in human keratinocytes.. Exp Dermatol.

[42] Vos JB, van Sterkenburg MA, Rabe KF, Schalkwijk J, Hiemstra PS (2005). Transcriptional response of bronchial epithelial cells to *Pseudomonas aeruginosa*: identification of early mediators of host defense.. Physiol Genomics.

[43] Higashimoto Y, Yamagata Y, Iwata T, Ishiguchi T, Okada M (2005). Adenoviral E1A suppresses secretory leukoprotease inhibitor and elafin secretion in human alveolar epithelial cells and bronchial epithelial cells.. Respiration.

[44] Bergman P, Johansson L, Asp V, Plant L, Gudmundsson GH (2005). *Neisseria gonorrhoeae* downregulates expression of the human antimicrobial peptide LL-37.. Cell Microbiol.

[45] Zhou L, Tan A, Hershenson MB (2004). Yersinia YopJ inhibits pro-inflammatory molecule expression in human bronchial epithelial cells.. Respir Physiol Neurobiol.

[46] Beisswenger C, Coyne CB, Shchepetov M, Weiser JN (2007). Role of p38 MAP kinase and transforming growth factor-beta signaling in transepithelial migration of invasive bacterial pathogens.. J Biol Chem.

[47] Wick M, Burger C, Funk M, Muller R (1995). Identification of a novel mitogen-inducible gene (mig-6): regulation during G1 progression and differentiation.. Exp Cell Res.

[48] Fiorentino L, Pertica C, Fiorini M, Talora C, Crescenzi M (2000). Inhibition of ErbB-2 mitogenic and transforming activity by RALT, a mitogen-induced signal transducer which binds to the ErbB-2 kinase domain.. Mol Cell Biol.

[49] Makkinje A, Quinn DA, Chen A, Cadilla CL, Force T (2000). Gene 33/Mig-6, a transcriptionally inducible adapter protein that binds GTP-Cdc42 and activates SAPK/JNK. A potential marker transcript for chronic pathologic conditions, such as diabetic nephropathy. Possible role in the response to persistent stress.. J Biol Chem.

[50] Monick MM, Cameron K, Staber J, Powers LS, Yarovinsky TO (2005). Activation of the epidermal growth factor receptor by respiratory syncytial virus results in increased inflammation and delayed apoptosis.. J Biol Chem.

[51] Zhang X, Pickin KA, Bose R, Jura N, Cole PA (2007). Inhibition of the EGF receptor by binding of MIG6 to an activating kinase domain interface.. Nature.

[52] O'Grady E, Mulcahy H, Adams C, Morrissey JP, O'Gara F (2007). Manipulation of host Kruppel-like factor (KLF) function by exotoxins from diverse bacterial pathogens.. Nat Rev Microbiol.

[53] Exley RM, Sim R, Goodwin L, Winterbotham M, Schneider MC (2009). Identification of meningococcal genes necessary for colonization of human upper airway tissue.. Infect Immun.

[54] O'Dwyer CA, Reddin K, Martin D, Taylor SC, Gorringe AR (2004). Expression of heterologous antigens in commensal *Neisseria* spp.: preservation of conformational epitopes with vaccine potential.. Infect Immun.

[55] Blake MS, MacDonald CM, Klugman KP (1989). Colony morphology of piliated *Neisseria meningitidis*.. J Exp Med.

[56] van Ulsen P, Tommassen J (2006). Protein secretion and secreted proteins in pathogenic *Neisseriaceae*.. FEMS Microbiol Rev.

[57] Virji M, Kayhty H, Ferguson DJ, Alexandrescu C, Heckels JE (1991). The role of pili in the interactions of pathogenic *Neisseria* with cultured human endothelial cells.. Mol Microbiol.

[58] Virji M, Makepeace K, Peak IR, Ferguson DJ, Jennings MP (1995). Opc- and pilus-dependent interactions of meningococci with human endothelial cells: molecular mechanisms and modulation by surface polysaccharides.. Mol Microbiol.

[59] Uronen-Hansson H, Steeghs L, Allen J, Dixon GL, Osman M (2004). Human dendritic cell activation by *Neisseria meningitidis*: phagocytosis depends on expression of lipooligosaccharide (LOS) by the bacteria and is required for optimal cytokine production.. Cell Microbiol.

[60] Gruenert DC, Finkbeiner WE, Widdicombe JH (1995). Culture and transformation of human airway epithelial cells.. Am J Physiol.

[61] Cozens AL, Yezzi MJ, Kunzelmann K, Ohrui T, Chin L (1994). CFTR expression and chloride secretion in polarized immortal human bronchial epithelial cells.. Am J Respir Cell Mol Biol.

[62] Li MS, Chow NY, Sinha S, Halliwell D, Finney M (2009). A *Neisseria meningitidis* NMB1966 mutant is impaired for invasion of respiratory epithelial cells, survival in human blood and for virulence in vivo.. Med Microbiol Immunol.

[63] Hartman AL, Ling L, Nichol ST, Hibberd ML (2008). Whole-genome expression profiling reveals that inhibition of host innate immune response pathways by Ebola virus can be reversed by a single amino acid change in the VP35 protein.. J Virol.

[64] Tusher VG, Tibshirani R, Chu G (2001). Significance analysis of microarrays applied to the ionizing radiation response.. Proc Natl Acad Sci U S A.

[65] Livak KJ, Schmittgen TD (2001). Analysis of relative gene expression data using real-time quantitative PCR and the 2(-Delta Delta C(T)) Method.. Methods.

[66] Mukhopadhyay TK (2008).

