# Supplementary material for: Genome Wide Expression Profiling Reveals Suppression of Host Defence Responses during Colonisation by Neisseria meningitides but not N. lactamica
Source: PLoS One. 2011 Oct 20;6(10):e26130. doi: 10.1371/journal.pone.0026130 (PMC3197596; doi:10.1371/journal.pone.0026130)
Supplement: Table S1 — Microarray and Q-RT-PCR results showing host gene expression fold changes from 5 to 7 hours in response to live WT N. lactamica and N. meningitidis. (DOC) [file pone.0026130.s001.doc]

**Table S1.** Microarray and Q-RT-PCR results showing host gene expression fold changes from 5 to 7 hours in response to live WT *N. lactamica* and *N. meningitidis*.

| **Human Genbank ID** | **Symbol** | **Description** | **Time/hour** | | | | | | | | | | | |
| --- | --- | --- | --- | --- | --- | --- | --- | --- | --- | --- | --- | --- | --- | --- |
|  |  |  | 5 | | | | 6 | | | | 7 | | | |
|  |  |  | *N. lactamica* | | *N. meningitidis* | | *N. lactamica* | | *N. meningitidis* | | *N. lactamica* | | *N. meningitidis* | |
|  |  |  | Array | Q-RT-PCR | Array | Q-RT-PCR | Array | Q-RT-PCR | Array | Q-RT-PCR | Array | Q-RT-PCR | Array | Q-RT-PCR |
| NM_003155 | STC1 | stanniocalcin 1 | 2.9 | 2.1 | 2.3 | 2.1 | 2.7 | 2.4 | 2.8 | 2.9 | 2.7 | 2.7 | 2.6 | 3.2 |
| NM_003714 | STC2 | stanniocalcin 2 | 3.1 | 2.9 | 3.1 | 3.1 | 3.3 | 3.4 | 3.5 | 3.8 | 3.7 | 4.6 | 2.4 | 3.1 |
| NM_001975 | ENO2 | enolase 2 | -- | -- | -- | -- | 3.1 | 2.9 | 2.7 | 4.3 | 4.6 | 3.7 | 3.3 | 3.9 |
| NM_000189 | HK2 | hexokinase 2 | -- | -- | -- | -- | 4.0 | 6.2 | 4.7 | 9.1 | 6.1 | 7.2 | 6.7 | 7.7 |
| NM_004566 | PFKFB3 | 6-phosphofructo-2-kinase 3 | -- | -- | -- | -- | 2.0 | 6.2 | 2.6 | 8.6 | 2.5 | 6.5 | 2.5 | 5.7 |
| NM_004567 | PFKFB4 | 6-phosphofructo-2-kinase 4 | -- | -- | -- | -- | 4.2 | 4.3 | 6.2 | 7.4 | 4.8 | 5.0 | 6.1 | 6.8 |

Regulation of phosphate metabolism process was over-represented from 5 to 7 hours and is represented by STC1 and STC2, while the glycolytic process was over-represented from 6 to 7 hours and is represented by ENO2, HK2, PFKFB3 and PFKFB4. The gene expression fold changes were compared to mock-infected controls.
